# *Pseudorhabdosynochus regius* n. sp. (Monogenea, Diplectanidae) from the mottled grouper *Mycteroperca rubra* (Teleostei) in the Mediterranean Sea and Eastern Atlantic

**DOI:** 10.1051/parasite/2015005

**Published:** 2015-02-13

**Authors:** Amira Chaabane, Lassad Neifar, Jean-Lou Justine

**Affiliations:** 1 Laboratoire de Biodiversité et Écosystèmes Aquatiques, Faculté des Sciences de Sfax, Université de Sfax BP 1171 3038 Sfax Tunisia; 2 Institut de Systématique, Évolution, Biodiversité, ISYEB, UMR 7205 – CNRS, MNHN, UPMC, EPHE, Muséum National d’Histoire Naturelle, Sorbonne Universités 57 rue Cuvier CP51 75231 Paris cedex 05 France

**Keywords:** Diplectanidae, Grouper, *Mycteroperca rubra*, Mediterranean Sea, Eastern Atlantic, ICZN Article 8.5

## Abstract

*Pseudorhabdosynochus regius* n. sp. is described from the gills of the mottled grouper *Mycteroperca rubra* caught off Senegal, Tunisia and Libya (type-locality: off Dakar, Senegal). The species is distinguished from its congeners by the structure of its sclerotised vagina (length 26–35 μm), which exhibits a trumpet in continuity with the primary canal, a straight primary canal, and primary and secondary chambers included in a common sclerotised mass along the primary canal. The species is also characterised by small squamodiscs (length 20–40 μm) with 10–11 rows of rodlets. Its closest relatives (based on the structure of the sclerotised vagina) are species mostly found in the Mediterranean Sea and parasites on species of *Mycteroperca*. A second species of *Pseudorhabdosynochus* Yamaguti, 1958 is reported from the same host and localities but not described. A list of diplectanids from groupers in the Mediterranean Sea is provided. We point out that a recent article was not compliant with the new Article 8.5.3 of the International Code of Zoological Nomenclature; for this reason, three species, *P. nhatrangensis* Dang, Bristow, Schander & Berland, 2013, *P. vietnamensis* Dang et al., 2013 and *P. brunei* Dang et al., 2013, are invalid.

## Abstract (fr)

*Pseudorhabdosynochus regius* n. sp. est décrit des branchies du mérou royal *Mycteroperca rubra* pêché au Sénégal, en Tunisie et en Libye (localité-type: au large de Dakar, Sénégal). L’espèce se distingue de ses congénères par la structure de son vagin sclérifié (longueur 26-35 μm), qui présente une trompette en continuité avec le canal primaire, un canal primaire droit, et des chambres primaire et secondaire incluses dans une masse sclérifiée commune le long du canal primaire. L’espèce est également caractérisée par de petits squamodisques (longueur 20-40 μm) avec 10-11 rangées de bâtonnets. Ses plus proches parents (sur la base de la structure du vagin sclérifié) sont pour la plupart des espèces trouvées dans la mer Méditerranée et parasites sur des espèces de *Mycteroperca*. Une seconde espèce de *Pseudorhabdosynochus* Yamaguti, 1958 est signalée dans le même hôte et même localités mais non décrite. Une liste des Diplectanidae de mérous en mer Méditerranée est fournie. Nous signalons qu’un article récent n’était pas conforme avec le nouvel article 8.5.3 du Code International de Nomenclature Zoologique; pour cette raison, trois espèces, *P. nhatrangensis* Dang, Bristow, Schander & Berland, 2013, *P. vietnamensis* Dang et al., 2013 et *P. brunei* Dang et al., 2013 ne sont pas valides.

## Introduction

The mottled grouper *Mycteroperca rubra* inhabits a rocky environment in the Mediterranean Sea and along the coast of the Eastern Atlantic Ocean [8]; it is common off Senegal but rare along the North African coast [8, 41]. Groupers generally harbour numerous diplectanid monogenean parasites [18, 19] and those from the Mediterranean Sea are no exception (Table 1).

**Table 1.** Groupers in the Mediterranean (according to [7, 8]) and their diplectanid species. The placement of certain host species in *Mycteroperca* follows recent molecular results [4, 40]; some of these species were previously classified within *Epinephelus.*

Host species	Diplectanid species, authorship of description and current combination, additional references
Native Mediterranean species	
*Epinephelus aeneus* (Geoffroy Saint-Hilaire)	*Pseudorhabdosynochus hargisi* (Oliver & Paperna, 1984) Santos, Buchmann & Gibson, 2000 [3, 36]. Redescription: [45]
	*P. americanus* (Price, 1937) Kritsky & Beverley-Burton, 1986 [25, 37]. Redescriptions: [6, 36, 45]
*Mycteroperca marginata* (Lowe, 1834) = *E. marginatus*	*P. riouxi* (Oliver, 1986) Santos, Buchmann & Gibson, 2000 [33, 38].
	Redescriptions: [34, 45]
	*P. beverleyburtonae* (Oliver, 1984) Kritsky & Beverley-Burton, 1986 [25, 32].
	Redescriptions: [35, 38]
	*Echinoplectanum echinophallus* (Euzet & Oliver, 1965) Justine & Euzet, 2006 [6, 21]
*M. costae* (Steindachner) *= E. costae*	*P. bouaini* Neifar & Euzet, 2007 [31]
	*P. dolicocolpos* Neifar & Euzet, 2007 [31]
	*P. enitsuji* Neifar & Euzet, 2007 [31]
	*P. sinediscus* Neifar & Euzet, 2007 [31]
	*P. sosia* Neifar & Euzet, 2007 [31]
*M. canina* (Valenciennes) *= E. caninus*	None recorded in the Mediterranean
*M. rubra* (Bloch)	*P. regius* n. sp. (this paper)
	*P.* sp. (this paper)
*Hyporthodus haifensis* (Ben-Tuvia)	*P.* sp. (unpublished)
Introduced Lessepsian species	
*E. coioides* (Hamilton)	None recorded in the Mediterranean; several diplectanids in its native range. South China Sea: [3, 27, 28, 45, 46]
*E. malabaricus* (Bloch & Schneider)	None recorded in the Mediterranean; several diplectanids in its native range. New Caledonia: [23]
*E. fasciatus* (Forsskål)	None recorded in the Mediterranean; several diplectanids in its native range. Red Sea: [36]; New Caledonia: [9, 14, 20]
Introduced Atlantic species	
*Cephalopholis taeniops* (Valenciennes)	None recorded in the Mediterranean (unidentified diplectanids present off Dakar, Senegal; unpublished)
*M. fusca* (Lowe)	None recorded in the Mediterranean
Introduced species – aquarium escapee	
*E. merra* (Bloch)	None recorded in the Mediterranean; several diplectanids in its native range. Fiji: [26]; New Caledonia: [9, 14]

We found two species of *Pseudorhabdosynochus* Yamaguti, 1958 on the gill filaments of *M. rubra* from Senegal, Tunisia and Libya; these are the first monogeneans reported from this fish. In this paper, we describe the most abundant of these species. The other species, which was rare, will be described when more material enables a full study.

## Materials and methods

Five *Mycteroperca rubra* were obtained from fish markets, including two specimens at Ouakam, Dakar, Senegal (February 2003), two at Sfax, Tunisia (January 2005) and one at Tripoli, Libya (June 2013). In all cases, the fish were dead, but, although their monogenean parasites were in suboptimal condition, they were considered suitable for study. The specimens collected from the fish gills were examined in Petri dishes containing seawater, using a stereomicroscope with incident light. These monogeneans were prepared according to three methods: (a) mounted in ammonium picrate-glycerine [29] (designated as “p” with regard to their measurements; (b) mounted in Berlese (designated “b”); (c) fixed unflattened in ethanol on the gills, then later rehydrated for examination, dehydrated in an ethanol series, stained with carmine, cleared with clove oil and mounted in Canada balsam (unflattened carmine, designated “uc”).

**Figure 1.**
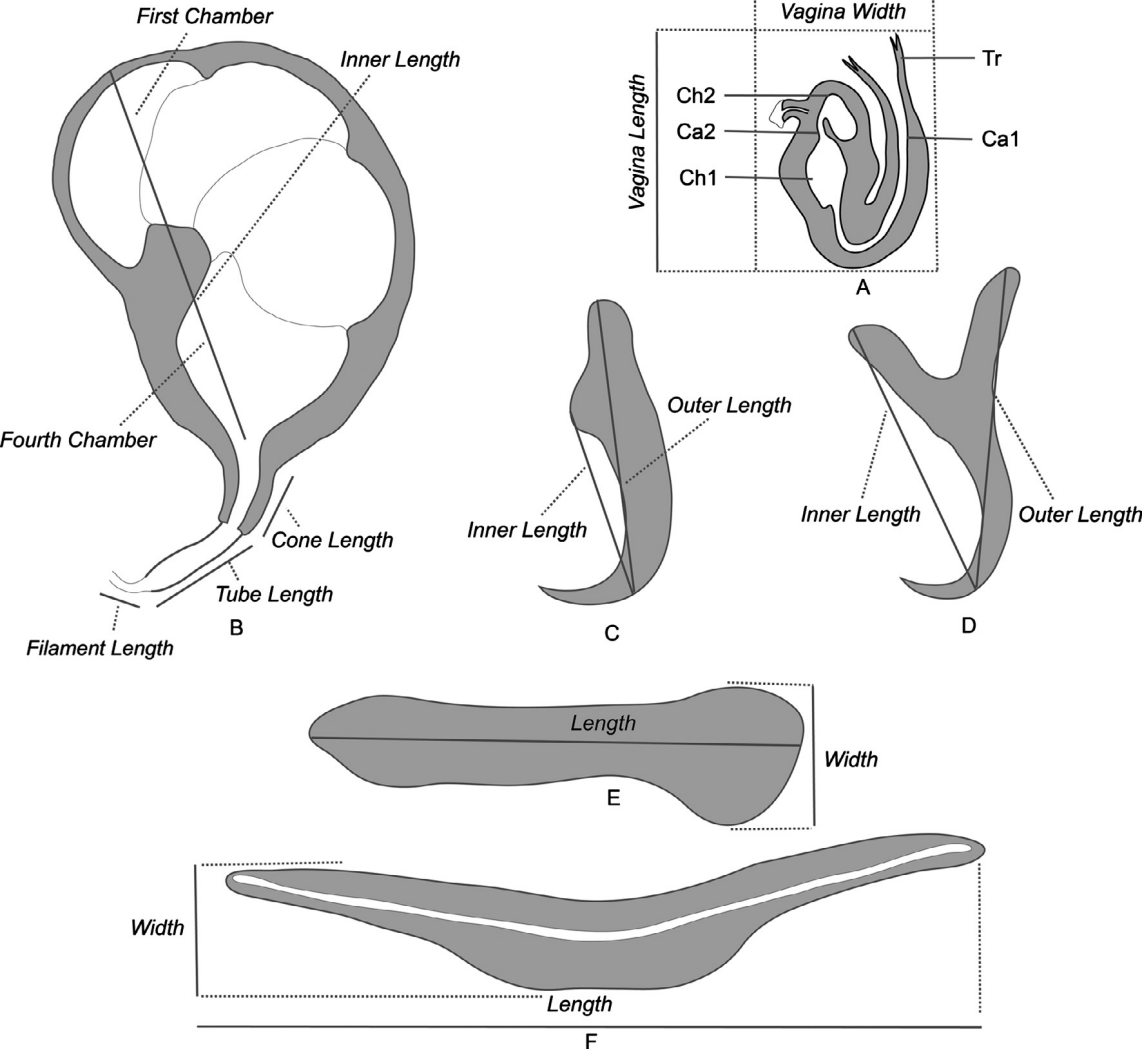
Methods of measurements and nomenclature of the sclerotised organs: A, sclerotised vagina: Tr, trumpet, Ca1, primary canal, Ch1, primary chamber, Ch2, secondary chamber, Ca2, secondary canal. B, male quadriloculate organ. C, ventral hamulus. D, dorsal hamulus. E, lateral (dorsal) bar. F, ventral bar.

Monogeneans were drawn with the aid of an Olympus BH2 microscope equipped with a drawing apparatus and DIC optics. The sclerotised parts were measured and designated according to Figure 1. The measurements of the right-hand haptoral hard-parts and left-hand equivalents were pooled. All measurements on the drawings were taken with the help of a custom-made transparent rule and are in micrometres. Measurements of the holotype (h) are given separately. Drawings were scanned and redrawn on a computer using Adobe Illustrator.

## *Pseudorhabdosynochus regius* n. sp.

urn:lsid:zoobank.org:act:19502FF9-544E-4364-B8D3-D139BB726BCC

Type-host: *Mycteroperca rubra* (Bloch) (Perciformes, Serranidae).

Site of infection: Gills.

Type-locality: Off Dakar (Senegal), February 2003.

Other localities: Off Sfax (Tunisia), January 2005; off Tripoli (Libya), June 2013.

Material examined: 43 specimens, including 5 “unflattened carmine” (uc), 8 “picrate” (p), 30 “berlese” (b).

Prevalence: 80%.

Type-specimens: Holotype and paratypes deposited in the Muséum national d’Histoire Naturelle, Paris (MNHN) as HEL516-517.

Etymology: The species name *regius* (a Latin adjective meaning “royal”) reflects the French name of the host, “Mérou royal”.

### Description (Figs. 2–3)

Body length h 860, uc 898 (600–1300, *n* = 4), p 824 (650–1000, *n* = 7), b 909 (650–1200, *n* = 10) including haptor; maximum width h 140, b 157 (150–170, *n* = 3) at level of ovary. Tegument smooth. Anterior region with three pairs of head organs and two pairs of eye-spots. External width of anterior eye-spot pair h 30, uc 34 (30–41, *n* = 4), p 38 (30–45, *n* = 7), b 35 (20–52, *n* = 18), of posterior pair h 23, uc 28 (23–38, *n* = 4), p 32 (25–43, *n* = 7), b 30 (15–38, *n* = 16). Pharynx median, subspherical, length h 52, uc 51 (45–55, *n* = 5), p 42 (32–50, *n* = 7), b 45 (33–55, *n* = 11), width h 52, uc 52 (50–55, *n* = 5), p 46 (38–53, *n* = 7), b 45 (32–60, *n* = 11). Haptor differentiated from the rest of body, width h 140, provided with 2 squamodiscs, 2 pairs of lateral hamuli, 3 bars and 14 marginal hooklets (7 pairs). Dorsal and ventral squamodiscs round with 10–11 rows of rodlets; 2–3 innermost rows V-shaped. Ventral squamodisc, length uc 34 (28–40, *n* = 2), p 27 (20–33, *n* = 2), b 25 (22–30, *n* = 3), width uc 31 (28–33, *n* = 2), p 27 (20–33, *n* = 2), b 21 (20–23, *n* = 3); dorsal squamodisc, length uc 32 (30–33, *n* = 2), b 33 (33–33, *n* = 2),width uc 25 (22–28, *n* = 2), b 25 (23–26, *n* = 2). Ventral hamulus with handle and distinct guard, outer length uc 44 (40–52, *n* = 6), p 45 (41–48, *n* = 16), b 46 (41–53, *n* = 36), inner length h 44, uc 40 (36–45, *n* = 9), p 42 (37–46, *n* = 16), b 42 (36–47, *n* = 39). Dorsal hamulus with indistinct guard, outer length uc 40 (38–42, *n* = 7), p 41 (39–43, *n* = 16), b 40 (22–45, *n* = 34), inner length uc 23 (22–24, *n* = 8), p 24 (22–25, *n* = 16), b 25 (22–66, *n* = 30). Lateral bar with wide flattened medial extremity and cylindrical lateral extremity, length h 46, uc 46 (43–53, *n* = 10), p 48 (44–50, *n* = 16), b 55 (44–70, *n* = 46), width h 15, uc 13 (10–16, *n* = 10), p 15 (12–20, *n* = 16), b 17 (12–22, *n* = 46).Ventral bar with small, constricted, median portion and blunt ends, length h 62, uc 62 (56–68, *n* = 5), p 65 (58–71, *n* = 8), b 71 (30–85, *n* = 23), width h 10, uc 10 (9–13, *n* = 5), p 10 (8–11, *n* = 8), b 13 (8–20, *n* = 23).

**Figure 2.**
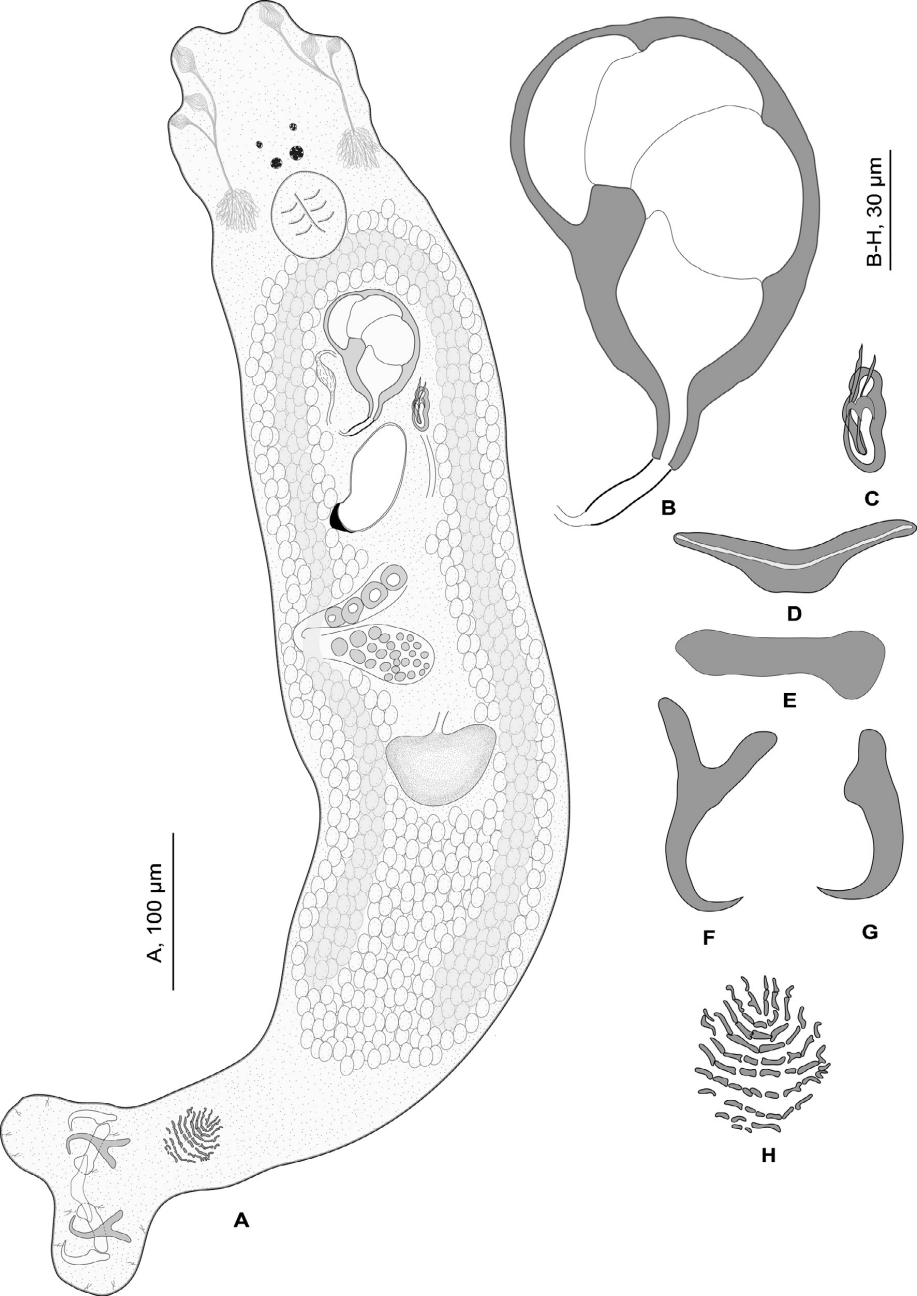
*Pseudorhabdosynochus regius* n. sp. from *Mycteroperca rubra*. A, composite (mainly from the holotype). B, male quadriloculate organ. C, sclerotised vagina. D, ventral bar. E, dorsal bar. F, ventral hamulus. G, dorsal hamulus. H, ventral squamodisc (paratype) A–H, carmine.

**Figure 3.**
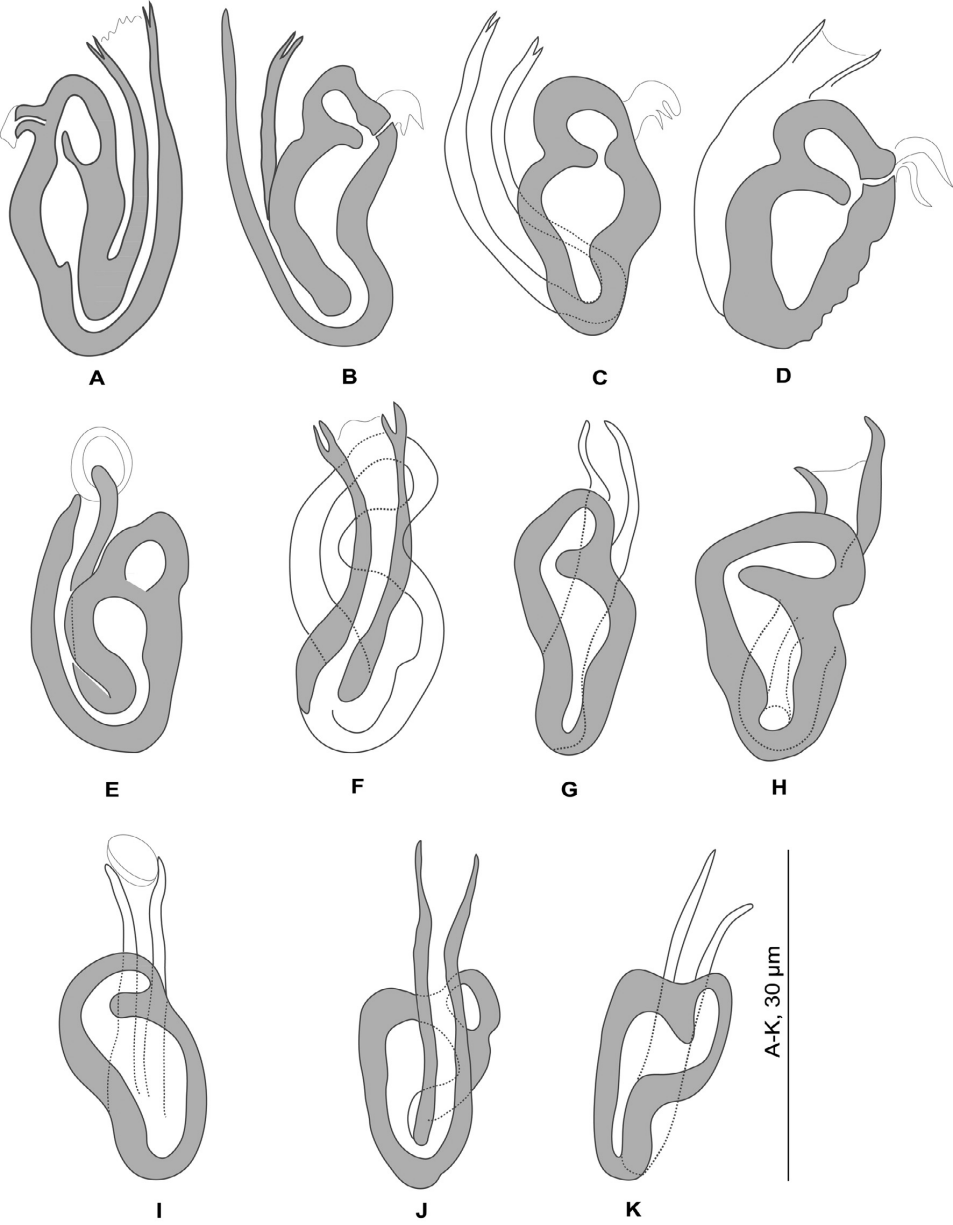
*Pseudorhabdosynochus regius* n. sp. from *Mycteroperca rubra*. Sclerotised vagina, variations according to different specimens, orientation and preparation. A-H, Berlese; I-K, carmine.

Testis subspherical, intercaecal. Quadriloculate organ with large sclerotised bulb divided into four chambers; internal length h 80, uc 77 (71–83, *n* = 5), p 98 (94–100, *n* = 4), b 85 (70–100, *n* = 25); fourth chamber ends in short sclerotised cone, length h 16, uc 16 (13–17, *n* = 4), p 13 (10–16, *n* = 5), b 16 (10–22, *n* = 25), prolonged by thin sclerotised tube; tube length h 21, uc 20 (18–21, *n* = 3), p 19 (18–20, *n* = 3), b 18 (15–20, *n* = 24); tube diameter h 4.5, uc 5 (4–5, *n* = 3), p 5 (4.5–5, *n* = 3), b 5 (4–5, *n* = 25); end of tube prolonged by short filament, not always visible, length h 10, uc 0–10 (*n* = 3), p 0–10 (*n* = 2), b 0–10 (*n* = 25).

Ovary dextral, loops dorsoventrally around right intestinal caecum. Vitelline follicles lateral, coextensive with intestinal caeca and contiguous posterior to testis. Egg inside genital tract, without filament, length uc 108–130 (*n* = 2), b 100–145 (*n* = 10).

*Sclerotised vagina* (nomenclature of parts according to Justine [15]) comprises anterior trumpet, primary canal, and distal sclerotised part, along primary canal, including both primary and secondary chambers. Trumpet in continuity with primary canal, with diameter slightly greater than canal. Primary canal straight, heavily sclerotised, curves just prior to entering primary chamber. Primary chamber heavily sclerotised, with its entrance posterior (i.e. its orientation is opposite to that of primary canal); secondary canal very short; secondary chamber, anterior to and smaller than primary chamber, with same heavily sclerotised structure. Accessory structure not seen. Sclerotised vagina length (measured from tip of trumpet to exterior of bend of primary canal) h 30, uc 29 (27–31, *n* = 5), p 31 (28–33, *n* = 7), b31 (26–35, *n* = 29).

### Differential diagnosis

The identification of species of *Pseudorhabdosynochus* is primarily based on the sclerotised vagina [14, 15, 17, 30]. However, the quadriloculate organ and the haptoral parts, including the squamodiscs, also provide characters useful for species identification [13, 14].

The general structure of the sclerotised vagina of *P. regius* n. sp. has the following characters: the trumpet in continuity with the linear primary canal, the orientation of the primary chamber opposite that of the primary canal, and the primary and secondary chambers grouped within a single heavily sclerotised structure along the primary canal. Other *Pseudorhabdosynochus* species which have a vaginal structure similar to *P. regius* are:

*Pseudorhabdosynochus sosia* Neifar & Euzet, 2007 (from *Mycteroperca costae*). This species can be differentiated by the shape of its trumpet (diameter similar to primary canal in *P. regius* vs. well differentiated in *P. sosia*), the shape of the anterior part of its primary canal (straight in *P. regius* vs. curved in *P. sosia*), the shape of its primary chamber and the length of the secondary canal (very short in *P. regius* vs. long in *P. sosia*). In addition, the two species have very different squamodiscs (central rows V-shaped in *P. regius*, circular in *P. sosia*).
*Pseudorhabdosynochus beverleyburtonae* (Oliver, 1984) Kritsky & Beverley-Burton, 1986 (from *M. marginata*). This species is close to *P. sosia* and can, therefore, be differentiated from *P. regius* based on the same characters.
*Pseudorhabdosynochus enitsuji* Neifar & Euzet, 2007 (from *M. costae*). Its sclerotised secondary chamber is larger than that in *P. regius*. In addition, the squamodiscs of *P. enitsuji* have numerous rows of rodlets (14–16).
*Pseudorhabdosynochus sinediscus* Neifar & Euzet, 2007 (from *M. costae*). The general structure is strikingly similar in this species, although the secondary chamber is not differentiated in the original description. *P. sinediscus* is differentiated from all other species of the genus, including *P. regius*, by the absence of squamodiscs.
*Pseudorhabdosynochus riouxi* (Oliver, 1986) Santos, Buchmann & Gibson, 2000 (from *M. marginata*). The general structure seems similar in this species, but the shape of the chambers (redescribed by Yang et al. [45]) is different. In addition, *P. riouxi* has squamodiscs with numerous rows of rodlets (11–22 in the original description).
*Pseudorhabdosynochus pai* Justine & Vignon, 2009 (from *E. tauvina* (Forsskål) in French Polynesian waters). The general structure is similar, but the primary canal is shorter in *P. regius* and the shape of the secondary chamber is different and complex (i.e. several secondary chambers) [24]. In addition, *P. pai* has a huge quadriloculate organ (72–144 inner length) and larger squamodiscs with numerous rows of rodlets (13–14). The hosts are also widely separated (Central Pacific vs. Atlantic/Mediterranean).

## Discussion

We describe a new species of *Pseudorhabdosynochus* from *Mycteroperca rubra* in this paper, but also found an additional species. Identification of the second species, which is close to *P. sosia* and *P. beverleyburtonae*, will require more material and the examination of comparative material.

Species of *Pseudorhabdosynochus* are mainly parasites of members of the family Epinephelidae (groupers), including species of *Epinephelus*, *Cephalopholis*, *Mycteroperca*, *Variola* and *Alphestes* [19, 30] but not *Plectropomus* [21]. A few species are parasites of members of the closely related Serranidae, including members of *Serranus* [44], *Paralabrax* [2], and of a member of the Polyprionidae [15]. A record on a member of the Chaetodontidae [1], never confirmed, is probably due to a mistake in the fish identification. Species of *Pseudorhabdosynochus* tend to be strictly host-specific, with species often restricted to a single host fish [19], but a few exceptions are known [17, 22, 30, 39]. An interesting aspect of our comparisons of *P. regius* with similar species, mainly based on the sclerotised vagina, is that most of the closely comparable species are parasitic on members of *Mycteroperca* (Table 1). This suggests that a group of *Pseudorhabdosynochus* species, with common vaginal characteristics, parasitises species of *Mycteroperca*, a genus which was found to be monophyletic in recent molecular studies [4, 40]. In contradiction to this hypothesis, *P. capurroi* Vidal-Martinez & Mendoza-Franco, 1998 from *M. bonaci*, *P. morrhua* Justine, 2008 and *P. variabilis* Justine, 2008, both from *M. morrhua*, do not share the same vaginal structure [16, 43]. Another hypothesis is that the three species of *Mycteroperca* with similar species of *Pseudorhabdosynochus* are all from the Mediterranean Sea (and Eastern Atlantic), suggesting that the close relationships of these parasite species reflect a common geographical origin, with the differentiation of various monogenean species in the groupers from the same area. However, a similar vaginal structure is found in *P. pai*, a parasite of *Epinephelus tauvina* in the Pacific [24] (i.e. neither a species of *Mycteroperca* nor Mediterranean), thus negating the two hypotheses mentioned above. We conclude that the relationships of the various species of *Pseudorhabdosynochus* in the world are complex and resistant to a simple analysis.

*M. rubra* is common off Senegal but rare along the North African coast [8, 41]. Mediterranean populations of groupers were affected by the last glacial period. The presence of the same *Pseudorhabdosynochus* species in specimens of *M. rubra* from the Mediterranean Sea and the Atlantic Ocean suggests a new model, which merits investigation using molecular methods.

According to Table 1, the biodiversity of diplectanids in groupers of the Mediterranean appears lower than that observed in groupers from warmer seas and coral reefs [9, 15, 23, 42], where the number of species can reach up to 12 per fish species. However, not all grouper species of the Mediterranean have been studied in detail in terms of their parasite fauna (Table 1).

## Nomenclatural validity of three recently described species of *Pseudorhabdosynochus*

While comparing our specimens with other species, our attention was drawn to a recent paper [5] describing new species of *Pseudorhabdosynochus* which we checked in terms of its nomenclatural validity.

The recent amendment [11, 12] of the International Code of Zoological Nomenclature [10] concerns electronic publication. According to Article 8.5, “to be considered published, a work issued and distributed electronically must:

8.5.1. have been issued after 2011,
8.5.2. state the date of publication in the work itself, and
8.5.3. be registered in the *Official Register of Zoological Nomenclature* (ZooBank) […] and contain evidence in the work itself that such registration has occurred [10].”

The descriptions of three new species of *Pseudorhabdosynochus* from groupers of Vietnam were published in 2013 [5]. The paper was published in the journal *International Journal of Aquatic Sciences*. The website of this journal (http://www.journal-aquaticscience.com) claims that it is an electronic publication (expressed as “e-journal”) and no mention of a printed version appears on the website or on the papers themselves. Therefore, the nomenclatural validity of the species described in this journal depends upon compliance with Article 8.5 of the Code, reproduced above. Articles 8.5.1 (after 2011) and 8.5.2 (date of publication indicated in the work itself, as 27 June 2013) are satisfied. However, Article 8.5.3 is *not* satisfied: there is no mention of a ZooBank number associated with this work. Article 8.5.3.3. describes the cases in which an error can be admitted: “An error in stating the evidence of registration does not make a work unavailable, provided that the work can be unambiguously associated with a record created in the *Official Register of Zoological Nomenclature* **before** the work was published” (the emboldened “before” is our own). We checked (2 December 2014) and found that neither these species nor the paper is indexed in ZooBank. According to the examples illustrating Article 8.5.3.3., *even if the registration in ZooBank was performed now or later*, the work would be unavailable.

Furthermore, to have its paper registered in ZooBank, the journal needs to meet the criteria enumerated in Articles 8.5.3.1 and 8.5.3.2. Article 8.5.3.1 requires “the name and Internet address of an organization other than the publisher that is intended to permanently archive the work in a manner that preserves the content and layout, and is capable of doing so”: we found no such repository mentioned on the journal website. This means that if the publisher closes its website, all papers will be lost. Article 8.5.3.2 requires “an ISSN for the journal containing the work”: the journal does mention an ISSN (2008–8019) but we could not retrieve this ISSN number from online systems such as WorldCat (http://www.worldcat.org/). Although these details do not specifically concern the case of the species dealt with in the present work, they show, more generally, that the journal itself (*International Journal of Aquatic Sciences*) could not publish a valid species according to the new ICZN Article 8.5.

Article 11 of the ICZN lists the criteria that make a name available. “Article 11. Requirements. To be available, a name or, where relevant, a nomenclatural act must satisfy the following provisions: 11.1. Publication. The name or nomenclatural act must have been published, in the meaning of Article 8, after 1757 [10].” In other words, a work which does not fulfil the criteria of Article 8 (especially, in the case of an electronic publication, Article 8.5. and its new amendment) does not satisfy Article 11 and thus is not *published,* and the new names of taxa in this work are not *available*.

To make things clearer, the paper itself [5] is “published” in terms of the general vocabulary used for publications, but the work is *not* published according to the International Code of Zoological Nomenclature [10]. The names of the three new species mentioned in the paper are *unavailable*, i.e. have not been *published* according to the International Code of Zoological Nomenclature. They are *not* valid and cannot be used in any publication which respects the ICZN – i.e. normally, all scientific journals.

Finally, we give here a list of the three names which are unavailable, for the reasons stated above: *Pseudorhabdosynochus nhatrangensis* Dang, Bristow, Schander & Berland, 2013; *P*. *vietnamensis* Dang et al., 2013; *P. brunei* Dang et al., 2013.

## Conflict of Interest

The Editor-in-Chief of Parasite is one of the authors of this manuscript. COPE (Committee on Publication Ethics, http://publicationethics.org), to which Parasite adheres, advises special treatment in these cases. In this case, the peer review process was handled by an Invited Editor, Dominique Vuitton.
